# High-Definition Map-Based Autonomous Vehicle Localization Using LiDAR Point Cloud Similarity Metrics: A Comparative Experimental Study †

**DOI:** 10.3390/s26134286

**Published:** 2026-07-06

**Authors:** Sai S. Reddy, Luis G. Jaimes, Onur Toker

**Affiliations:** 1 Department of Computer Science, Florida Polytechnic University, Lakeland, FL 33805, USA; sreddyk7069@floridapoly.edu; 2 Department of Electrical & Computer Engineering, Florida Polytechnic University, Lakeland, FL 33805, USA; otoker@floridapoly.edu

**Keywords:** autonomous vehicles, point clouds, high-definition maps

## Abstract

Accurate localization is a critical requirement for autonomous vehicle (AV) navigation, particularly in environments where GPS signals are unreliable or unavailable. A wide range of LiDAR-based point cloud similarity metrics have been proposed for high-definition (HD) map localization, but systematic comparisons of distinct metric families on the same real-world dataset, under identical conditions, remain scarce. In this paper, we present an offline comparative study of three point cloud similarity metrics within a unified HD map-based localization framework, under the assumption of largely static environments and planar, yaw-dominant vehicle motion typical of on-road driving. The HD map is constructed as a directed graph of GPS coordinates, each linked to a corresponding LiDAR scan, collected over a 30-min drive on a university campus using a Velodyne VLP-16 sensor and a ublox ZED-F9P RTK-GPS receiver, yielding 19,500 time-synchronized point clouds. Within this framework, we develop and compare three similarity metrics drawn from distinct families: Fast Point Feature Histograms (FPFH) with KDTree-based matching, Procrustes-based alignment via singular value decomposition, and a planar projection method based on 2D angular histogram cross-correlation. Each metric is evaluated on the same dataset in terms of similarity score profile (localizability) and per-pair computational cost. FPFH provides rich local geometric matching but at an average per-pair cost of approximately 1018 s, making it suitable only for offline analysis. Procrustes alignment yields the smoothest score profiles, with an exact self-similarity baseline of zero, at an average of 2.63 s per pair. The planar projection method produces the most location-invariant profiles at an average of 11.6 s per pair. We also discuss the recursive localization architecture into which any of these metrics could be embedded, and analyze the gap between current per-pair costs and what would be required for online deployment, which we identify as a direction for future work. The study contributes a controlled, reproducible benchmark of three metric families on a single real-world dataset, and provides guidance for selecting similarity metrics under stated operating assumptions.

## 1. Introduction

Autonomous vehicles (AVs) rely on accurate and reliable localization to navigate safely in complex environments. Modern AV systems typically combine multiple sensors, including cameras, radar, and LiDAR, alongside GPS receivers, to determine their position in real time [1]. However, GPS signals are frequently degraded or entirely unavailable in challenging scenarios such as urban canyons with high-rise buildings, tunnels, dense forests, and indoor environments. In these GPS-denied or GPS-degraded conditions, traditional localization methods that depend heavily on satellite signals fail to provide the centimeter-level accuracy required for safe autonomous navigation. This limitation represents one of the most pressing open problems in the field of autonomous vehicle research.

LiDAR sensors have emerged as a particularly promising technology for addressing this challenge. Unlike cameras, which are sensitive to lighting conditions, and GPS, which is vulnerable to signal obstruction, LiDAR generates dense, accurate three-dimensional point clouds of the surrounding environment under a wide range of conditions [1]. These point clouds capture the geometric structure of the environment in fine detail, making them well-suited for localization tasks based on matching current sensor observations against a pre-built map. High-definition (HD) maps have, in turn, emerged as a powerful complement to LiDAR-based perception in GPS-denied environments [2]. An HD map provides a detailed representation of the environment encoded at centimeter-level precision, far exceeding the accuracy of conventional navigation maps. In the context of LiDAR-based localization, an HD map can be constructed as a directed graph of GPS coordinates, with each node linked to a corresponding LiDAR scan collected at that location [3]. Given such a map, the localization problem reduces to finding the map entry whose stored point cloud most closely matches the point cloud currently observed by the vehicle’s LiDAR sensor. The effectiveness of this approach depends critically on the choice of similarity metric used to compare point clouds, since different metrics offer different trade-offs between matching behavior, robustness to noise, and computational cost [4].

Despite a growing body of literature on point cloud similarity and LiDAR-based localization, a systematic experimental comparison of distinct similarity metric families, evaluated under identical real-world conditions on the same dataset, remains largely absent. Feature-based methods such as Fast Point Feature Histograms (FPFH) have been widely studied for their ability to capture rich local geometric structure [5], but their computational cost is substantial. Global alignment methods such as Procrustes analysis offer rotation and translation invariance through efficient matrix operations, yet their behavior in real-world localization scenarios has received comparatively little attention. Projection-based methods, which reduce three-dimensional point clouds to two-dimensional representations for efficient comparison, have shown promise in recent work [6,7], but have not been rigorously benchmarked against the other two families under controlled experimental conditions. This gap motivates the present study.

In this paper, we propose a unified HD map-based localization framework and evaluate three point cloud similarity metrics within it. At a high level, the framework consists of three components: an HD map, organized as a directed graph of GPS-tagged LiDAR scans collected from a real driving session; a similarity oracle, instantiated by any one of the three metrics under study; and a recursive update procedure that, given a known initial location, uses successive LiDAR scans together with the similarity oracle to track the vehicle’s position over time. The role of the present study is not to deploy this framework as an online system, but to characterize the behavior and cost of each similarity oracle on the same real-world data, so that informed metric selection becomes possible for any future deployment of the framework. The dataset used throughout the paper was collected from an autonomous vehicle research platform equipped with a Velodyne VLP-16 LiDAR sensor (Ouster, San Jose CA, USA) and a ublox ZED-F9P RTK-GPS receiver (u-blox AG, Thalwil, Switzerland), driven across a university campus for over 30 min, yielding 19,500 synchronized point clouds and GPS measurements [8,9,10]. The three similarity methods evaluated are FPFH with KDTree-based nearest neighbor matching [5], Procrustes-based alignment using singular value decomposition, and a planar projection method based on two-dimensional histogram cross-correlation computed via Fast Fourier Transform techniques [10].

It is important to state, from the outset, the scope and operating assumptions under which the framework and the comparative results in this paper should be interpreted. First, the study is an offline comparative analysis. The per-pair computation times reported in Section 6 are substantially larger than the one-second update cycle that an online implementation of the recursive framework would require, and we therefore do not claim real-time deployability for any of the three metrics in their present form. The recursive update procedure is presented as the intended deployment architecture into which a future, optimized similarity oracle could be embedded, rather than as a system that runs online today. Second, the methods assume planar, yaw-dominant vehicle motion typical of on-road driving. The minimization over the three-dimensional rotation group SO(3) that defines the ideal similarity between two point clouds is, throughout this work, approximated by a search over yaw and a complementary angular component, which is consistent with the assumption that pitch and roll vary only slightly between nearby scans on a road surface. The mathematical formulations in Section 4, and in particular the equivalence between three-dimensional rotation and circular shift in the angular histogram used by the planar projection method, should be read under this restricted motion model. Third, the HD map is assumed to be largely static and to reflect the environment as it was during the data collection drive. The recursive algorithm also assumes that an initial GPS fix is available; cold-start localization without any prior position estimate is outside the scope of this study. Sensitivity of the methods to dynamic environmental changes, adverse weather, and trajectories that violate the planar motion assumption are discussed as limitations in Section 7.3 and as directions for future work in Section 8.2.

Within this stated scope, the contributions of this work are as follows. First, we formulate a unified HD map-based localization framework in which the HD map, the similarity oracle, and the recursive update procedure are described as three clearly separated components, allowing any point cloud similarity metric to be plugged in as the oracle without altering the rest of the system. Second, we implement three similarity metrics drawn from three distinct families, namely feature-based (FPFH with KDTree), global alignment (Procrustes via singular value decomposition), and projection-based (planar angular-histogram cross-correlation), within this common framework. Third, we conduct a controlled experimental comparison of these three metrics on the same real-world dataset of 19,500 scans, characterizing both the localizability of each metric, in the form of its similarity score profile around a reference frame, and its per-pair computational cost over five independent trials. Fourth, we analyze the gap between current per-pair costs and the requirements of online operation under the recursive framework, and identify the algorithmic and hardware directions that would be necessary to close that gap. Together, these contributions provide a reproducible benchmark of three metric families on a single real-world dataset and offer principled guidance for selecting similarity metrics under the operating assumptions stated above.

The remainder of this paper is organized as follows. Section 2 reviews the relevant literature on map-based localization, point cloud similarity, and LiDAR-based place recognition. Section 3 describes the data collection process and HD map construction. Section 4 presents the three point cloud similarity methods and their mathematical formulations under the planar, yaw-dominant motion model. Section 5 describes the recursive localization framework and clarifies how each similarity oracle integrates into it. Section 6 presents the experimental results, including similarity score profiles and computational time comparisons. Section 7 discusses the findings, the trade-offs across metrics, and the limitations of the present study. Section 8 concludes the paper and outlines directions for future work, including the algorithmic and hardware steps that would be required to bring the framework toward online operation.

## 2. Related Work

The problem of LiDAR-based localization for autonomous vehicles has attracted considerable research attention over the past decade. Existing approaches can be broadly categorized into map-based localization methods, feature-based similarity techniques, projection and descriptor-based approaches, and more recently, deep learning frameworks. In this section, we review the most relevant work in each of these categories, highlighting their contributions, limitations, and relationship to the present study.

### 2.1. Map-Based Localization and High-Definition Maps

Map-based localization has become one of the dominant paradigms for achieving robust and accurate vehicle positioning in GPS-denied environments. The core idea is to match sensor observations against a pre-built representation of the environment, allowing the vehicle to determine its position even in the absence of reliable satellite signals. Chalvatzaras et al. [4] provide a comprehensive survey of map-based localization techniques for autonomous vehicles, categorizing existing approaches into metric-based, feature-based, topological, and hybrid methods. Their work emphasizes the critical role of LiDAR sensors in achieving high-precision localization and identifies the ongoing challenge of balancing accuracy, scalability, and computational cost in real-world deployments. These observations directly motivate the present study, which seeks to identify which similarity metric family offers the most favorable trade-off under identical experimental conditions.

The structure and content of HD maps play a central role in map-based localization pipelines. Liu et al. [2] provide a detailed overview of HD maps for automated driving, analyzing their structural composition, data layers, and semantic annotations. The authors emphasize that HD maps offer centimeter-level accuracy that far surpasses conventional GPS and IMU systems, particularly in complex urban environments. They also highlight the dependency of map-based localization on the fidelity and freshness of the stored map data, noting that outdated or incomplete maps can significantly degrade localization performance. This finding reinforces the importance of constructing maps from high-quality, time-synchronized sensor data, as done in the present work using a Velodyne VLP-16 LiDAR sensor and a ublox ZED-F9P RTK-GPS receiver. A more recent and comprehensive survey of HD-map construction, maintenance, and localization challenges is provided by Elghazaly et al. [11], who characterize the HD map as a virtual sensor that aggregates knowledge from physical sensors to provide a strong prior for automated vehicles.

Zang et al. [3] propose an accurate self-localization framework that leverages HD map datasets to improve vehicle positioning in autonomous navigation systems. Their method integrates LiDAR point cloud data with geometric features extracted from HD maps, enabling precise alignment between observed data and pre-mapped environments. The authors emphasize the importance of multi-level feature extraction, including curbs, road markings, and structural boundaries, and demonstrate how matching these elements can significantly enhance localization accuracy in urban environments where GPS is unreliable. This work aligns closely with the motivation behind the similarity-based methods explored in the present study, where structural matching between live LiDAR scans and stored map entries is central to the localization process.

Toker [8] presents an experimental performance analysis of a self-driving vehicle using HD maps, evaluating how map-based localization impacts route-following accuracy and system reliability under real-world driving conditions. The results show that even minor mismatches in map registration can lead to significant navigational errors, underscoring the importance of robust and accurate similarity metrics. The AV research platform used in that study is the same platform employed in the present work, providing a direct experimental continuity between the two contributions.

### 2.2. Feature-Based Methods

Feature-based methods approach the point cloud similarity problem by extracting compact descriptors that capture the local geometric structure around each point, and then comparing these descriptors across scans. Rusu et al. [5] introduced the Fast Point Feature Histogram (FPFH), a local 3D descriptor that significantly improves upon the computational efficiency of the earlier Point Feature Histogram (PFH). FPFH reduces the computational complexity of descriptor computation to O(k) by first computing a Simplified PFH (SPFH) for each point considering only its immediate neighbors, and then aggregating the SPFH values of neighboring points weighted by their spatial proximity. The authors also introduced the Sample Consensus Initial Alignment (SAC-IA) algorithm, which uses FPFH descriptors to achieve robust initial alignment of point clouds, facilitating convergence in subsequent fine registration stages. FPFH has since become one of the most widely adopted descriptors in point cloud registration and localization pipelines, and it forms one of the three similarity methods evaluated in the present study.

Huang and You [12] introduced a complementary approach for point cloud matching that leverages the concept of 3D self-similarity to establish robust correspondences between LiDAR scans. Rather than relying solely on local geometric descriptors or global alignment, their technique detects recurring structural patterns within the same point cloud and uses these patterns to identify similar regions across different scans. This self-similarity-driven framework demonstrates resilience to noise, partial occlusions, and varying point densities, making it particularly useful in unstructured or cluttered environments. While computationally intensive, this approach paved the way for alternative matching paradigms that emphasize relational rather than absolute geometry, complementing descriptor-based techniques such as FPFH.

The Iterative Closest Point (ICP) algorithm [13] represents another widely studied family of feature-based alignment methods. ICP iteratively minimizes the distance between corresponding points in two point clouds by alternating between correspondence search and transformation estimation. While effective for fine registration, ICP is sensitive to initialization and can converge to local minima, limiting its applicability in scenarios where the initial pose offset is large. Procrustes analysis, evaluated in the present study, is related in spirit to ICP in that both seek a rigid transformation that minimizes a residual error, but differs in that it computes the transformation in closed form via singular value decomposition, given a fixed point set rather than iteratively refining correspondences. As discussed in Section 4.3, the Procrustes formulation used here operates on point clouds of normalized cardinality and should be understood as an approximation rather than as a classical Procrustes alignment with explicit ordered correspondences.

### 2.3. Projection and Descriptor-Based Methods

Projection-based methods address the computational challenges of full 3D point cloud comparison by reducing the dimensionality of the data prior to matching. These approaches typically project the three-dimensional point cloud onto a two-dimensional representation, such as a range image or an angular histogram, and then apply efficient image-based comparison techniques.

Chen et al. [6] proposed a range image-based LiDAR localization approach tailored for autonomous vehicles, demonstrating improved robustness and efficiency in urban environments. By transforming raw LiDAR point clouds into range images, their method leverages two-dimensional convolutional neural networks for scan matching and pose estimation, significantly reducing computational complexity while preserving spatial structure. The proposed system demonstrated strong localization accuracy on challenging benchmarks including the KITTI and NCLT datasets. This work highlights the potential of image-like LiDAR representations for real-time autonomous navigation and provides an important precedent for the planar projection method proposed in the present study.

Kim and Kim [7] introduced Scan Context, an egocentric spatial descriptor for place recognition using 3D LiDAR point clouds. Their method projects point clouds into a cylindrical image representation, preserving relative spatial context while remaining invariant to sensor height and robust against rotation around the vertical axis. Scan Context captures global environmental features in a compact two-dimensional matrix, enabling efficient and scalable place matching with fast nearest-neighbor search and loop closure detection. The descriptor’s design and its use of a matrix-based representation share conceptual similarities with the planar projection method evaluated in this study, where a two-dimensional angular histogram is used as a compact representation of the three-dimensional point cloud for cross-correlation-based similarity scoring.

Reddy et al. [10] present a directly related experimental framework for 3D map generation and localization by merging RTK-GPS data with LiDAR point clouds. A key contribution of that study is the introduction of the planar projection-based similarity method, which reduces 3D point cloud complexity by projecting it onto a 2D angular histogram for comparison via FFT-based cross-correlation. The paper demonstrates that this representation retains sufficient structural information for similarity-based localization while substantially lowering the per-pair computational cost compared to a direct Chamfer-distance formulation with an explicit search over rotations. The present study extends that prior work by placing the planar projection method within a unified comparative framework alongside FPFH and Procrustes-based similarity, evaluated on the same dataset under identical conditions, so that the trade-offs across the three metric families can be characterized in a controlled manner.

### 2.4. Deep Learning Approaches

Recent years have seen a rapid growth in deep learning-based methods for LiDAR place recognition and localization. While these approaches are not the focus of the present study, they represent an important direction for future work and provide useful context for situating the contributions of the methods evaluated here.

Uy and Lee [14] proposed PointNetVLAD, a deep learning-based framework for large-scale place recognition using raw 3D point clouds. Their method combines the feature extraction capabilities of PointNet with the Vector of Locally Aggregated Descriptors (VLAD) layer to produce discriminative and compact global descriptors for place retrieval. Unlike traditional handcrafted features, PointNetVLAD operates directly on unordered point sets and learns spatial patterns in a data-driven manner, demonstrating superior performance on benchmark datasets such as the Oxford RobotCar.

Liu et al. [15] proposed LPD-Net, a deep learning architecture designed for large-scale place recognition using 3D point clouds. LPD-Net extracts local geometric features and aggregates them using a graph-based neighborhood topology that captures both spatial distribution and structural relationships, demonstrating robustness to viewpoint changes, occlusion, and environmental variability on multiple benchmark datasets.

Tinchev et al. [16] introduced a learning-based approach for place recognition using 3D LiDAR maps, employing a neural network trained with triplet loss to learn compact and discriminative descriptors from raw point clouds. Sun et al. [17] presented a two-stage pipeline that combines deep learning-based place recognition with precise pose estimation using point cloud registration, achieving strong localization performance in large-scale indoor and outdoor environments. Shi et al. [18] provide a comprehensive survey of LiDAR-based place recognition techniques for autonomous driving, cataloguing the evolution from early geometric descriptors to recent deep learning models and identifying key open challenges including rotational invariance, robustness to viewpoint changes, and computational efficiency.

While deep learning methods have demonstrated impressive results on standard benchmarks, they typically require large annotated training datasets, significant computational resources for both training and inference, and careful tuning for deployment in new environments. The methods evaluated in the present study, by contrast, are entirely training-free and rely only on the geometric structure of the point clouds themselves, making them immediately deployable on new environments without any prior learning phase. Investigating whether learning-based approaches can be integrated into the HD map localization framework proposed here, while preserving the real-time performance requirements of AV systems, remains an important direction for future work.

### 2.5. Summary and Position of the Present Study

Across the four categories surveyed above, two patterns emerge. First, each family of similarity metrics, feature-based, global-alignment, projection-based, and deep-learning, has been developed and evaluated largely within its own benchmark conventions, on datasets and under experimental protocols that differ from one another. Second, comparisons that do appear in the literature are typically internal to a family, for example one feature descriptor against another, or one learned model against another, rather than across families. As a result, a practitioner who must select a similarity metric for an HD map-based localization framework cannot straightforwardly determine, from existing publications alone, how a feature-based method, a global-alignment method, and a projection-based method would behave on the same data under the same operating assumptions. The present study addresses this gap by implementing one representative method from each of the three training-free families within a single framework, evaluating them on the same 19,500-scan real-world dataset, and characterizing both their similarity score profiles and their per-pair computational costs under the stated assumptions of planar yaw-dominant motion, a largely static map, and a known initial GPS fix. The deep-learning family is intentionally excluded from the experimental comparison, since the training requirements and data-driven assumptions of those methods place them in a different evaluation regime, and is instead discussed as a direction for future work in Section 8.2.

## 3. Data Collection and HD Map Construction

This section describes the autonomous vehicle research platform used in this study, the data collection protocol followed during campus driving, the clock synchronization procedure, and the construction of the high-definition map that serves as the reference database for all localization experiments.

### 3.1. Autonomous Vehicle Research Platform

The experimental data used in this study were collected using an autonomous vehicle research platform developed at Florida Polytechnic University. The platform is based on an electric golf cart converted to drive-by-wire operation, enabling full software control of steering, throttle, and braking [9]. The vehicle is equipped with a suite of sensors, of which two are directly relevant to the present work: a Velodyne VLP-16 LiDAR sensor and a ublox ZED-F9P RTK-GPS receiver mounted on a C099-F9P application board.

The Velodyne VLP-16 is a 16-channel LiDAR sensor that emits laser pulses at 16 different polar angles simultaneously, producing a full 360-degree scan of the surrounding environment. In this study, the sensor was configured to operate at a frame rate of 10 frames per second, meaning that a complete 360-degree scan was completed every 0.1 s. The resulting point clouds were visualized and post-processed using VeloView (version 5.1), a software package provided by Velodyne, which was used to remove noise, align scans, and export usable three-dimensional data in .ply format for further analysis. Three-dimensional visualizations were subsequently generated using the Python Open3D library (version 0.19).

The ublox ZED-F9P is a high-precision RTK-GPS receiver capable of centimeter-level positioning accuracy under favorable signal conditions. In this study, the RTK-GPS output rate was set to 2 Hz, and the Florida Department of Transportation’s Florida Primary Reference Network was used as the source of real-time correction signals. The GPS output follows the NMEA 0183 standard text format, which can be saved in KMZ format for visualization in Google Earth.

### 3.2. Campus Drive Protocol and Dataset

Data collection was performed by driving the AV research platform continuously across the Florida Polytechnic University campus in Lakeland, Florida, for approximately 1950 s, a little over half an hour. The starting point of the route was located close to the newly constructed BARC Applied Research Center.

**Figure 1:**
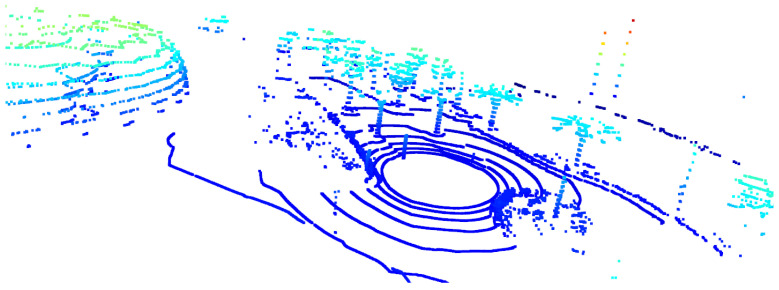
Point cloud obtained from the Velodyne VLP-16 LiDAR sensor at the entrance of the IST building, Florida Polytechnic University. The cloud consists of 18,165 three-dimensional points, visualized using the Python Open3D library. Colors represent different height values.

**Figure 2:**
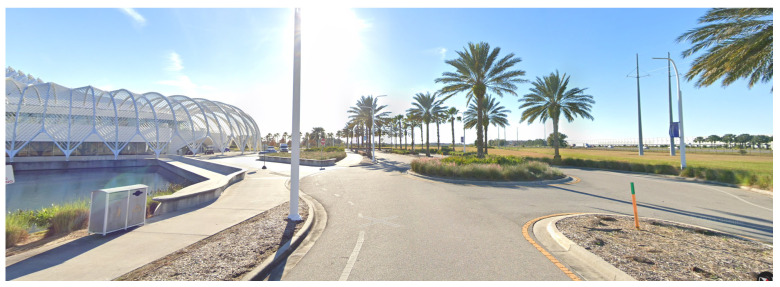
Google Street View of a nearby location corresponding to the point cloud shown in Figure 1, illustrating the physical environment captured by the LiDAR sensor.

A representative point cloud captured during the drive is shown in Figure 1. This particular scan was acquired at the entrance of the IST building on campus and consists of 18,165 three-dimensional vectors, stored as an 18,165×3 real-valued matrix. The Google Street View of the same location is shown in Figure 2 for reference, illustrating the correspondence between the point cloud representation and the physical environment.

**Figure 3:**
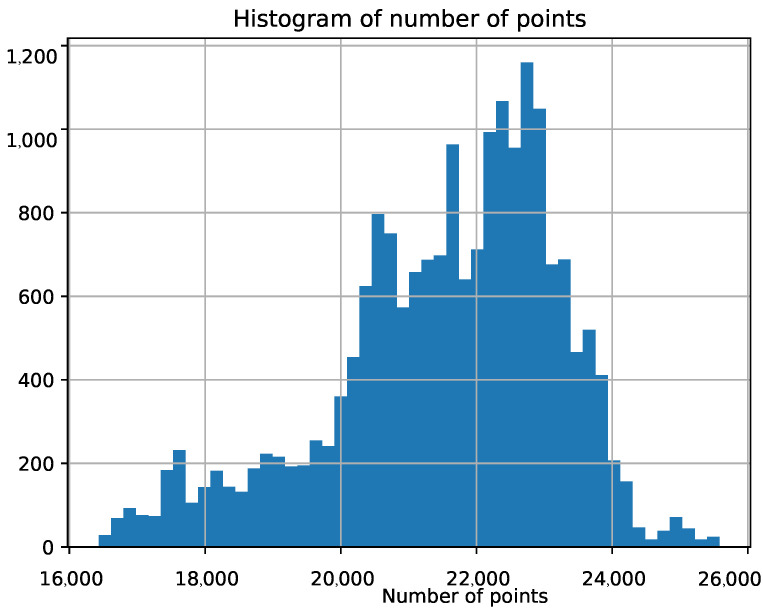
Histogram of the number of points per scan across the full dataset of 19,500 LiDAR point clouds. The mean is 21,533 points with a standard deviation of 1685 points.

During the drive, the LiDAR sensor captured data continuously at 10 fps, while the RTK-GPS receiver recorded position fixes at 2 Hz, yielding a total of N=19,500 LiDAR point clouds. A histogram of the number of points per scan across the full dataset is shown in Figure 3. The distribution has a mean of 21,533 points and a standard deviation of 1685 points, reflecting the natural variation in scene complexity encountered along the driven route.

### 3.3. Clock Synchronization and Data Interpolation

Since the LiDAR sensor operates at 10 fps and the RTK-GPS receiver operates at 2 Hz, the two data streams were recorded at different rates and required synchronization before the paired dataset could be used. The clocks of the two sensors were first synchronized, and the RTK-GPS data stream was then upsampled by a factor of five using linear interpolation, bringing it to a 10 fps rate that matched the LiDAR frame rate. Hardware-level synchronization between the two devices, such as driving a synchronization input on the LiDAR from the pulse-per-second (PPS) signal of the RTK-GPS module, was not employed in this data collection campaign; the use of software alignment followed by interpolation is the reason the GPS stream required upsampling rather than already being sample-aligned with the LiDAR. Linear interpolation is adequate at this scale because, at the operating speed of the research vehicle, the vehicle displacement between two consecutive 2 Hz GPS samples is small relative to the route geometry, and the interpolated positions remain consistent with the centimeter-level precision of the RTK-GPS receiver. This procedure ensured that every LiDAR point cloud $P_{k}$ in the dataset was associated with a unique and temporally consistent GPS location tag $g_{k}\in R^{3}$, forming the complete paired dataset

(1) $$P_{k},g_{k}\;k=0,\cdots ,N-1,$$

where N=19,500, the $P_{k}$’s are the point clouds, and the $g_{k}$’s are the location tags as three-dimensional vectors. We note that the dataset was collected and synchronized for offline analysis; the recursive localization framework described in Section 5 operates on this paired dataset post hoc and does not require real-time synchronization of the two streams during data collection.

**Figure 4:**
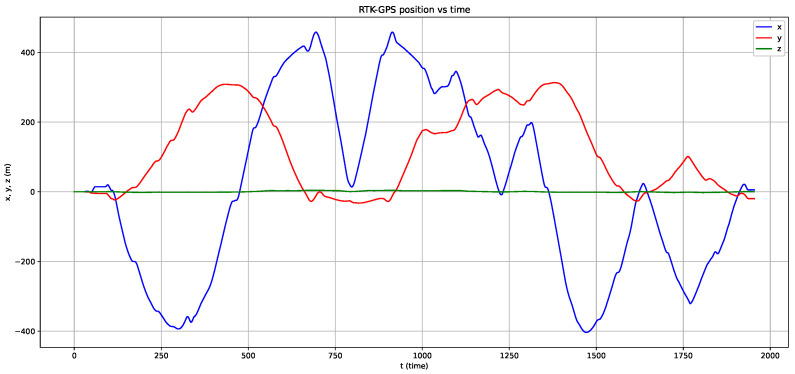
The path followed by the AV research vehicle expressed in Cartesian coordinates, i.e., (x(t),y(t),z(t)) versus *t*. The origin is a point close to the BARC Applied Research Center. The *x* (blue), *y* (red), and *z* (green) components are shown as a function of time over the full 1950-s drive.

**Figure 5:**
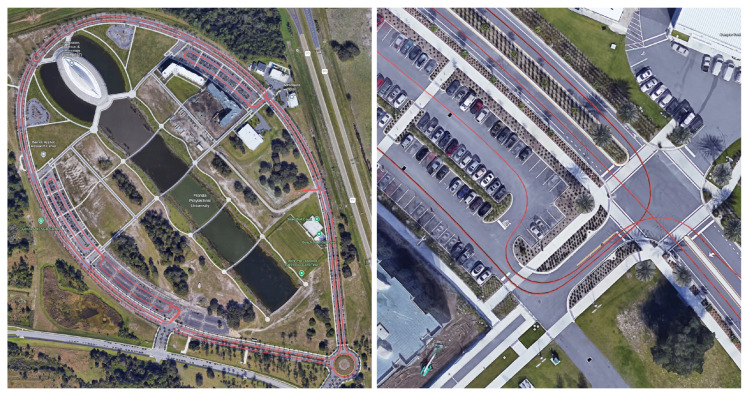
The path followed by the AV research vehicle shown in Google Earth (Red colored curves). The left panel shows the full campus route, and the right panel provides a zoomed view of a parking area section of the route.

The path followed by the AV research vehicle is shown in Cartesian coordinates in Figure 4, with the origin placed at a point close to the BARC Applied Research Center. The same path viewed from above in Google Earth is shown in Figure 5, illustrating the full extent of the campus route covered during data collection.

### 3.4. HD Map Construction

The synchronized dataset of point cloud and GPS pairs was used to construct a high-definition map of the campus environment. A point cloud is formally defined as a finite set of three-dimensional points,

(2) $$P=\{x_{k}\;:\;x_{k}\in R^{3},\;k=0,\cdots ,N-1\},$$

where the set is unordered, meaning that any permutation of the points $x_{k}$ results in the same point cloud object. Even when points are stored in an array-like data structure with integer indices, the point cloud is treated as a set throughout this work.

The HD map is defined as a directed graph in which each node represents a GPS coordinate $g_{k}\in R^{3}$ and is linked to its corresponding LiDAR point cloud $P_{k}$. This structure captures the spatial layout of the environment along the driven route and provides the reference database against which LiDAR scans are compared in the similarity studies of Section 4 and in the recursive localization framework of Section 5. The directed graph structure is also extensible, meaning that additional sensor modalities such as camera images or radar returns could be associated with each node to enrich the map representation in future work [2,8].

## 4. Point Cloud Similarity Methods

In autonomous vehicle localization, the ability to match a current LiDAR scan against entries in a pre-built high-definition map is critical for determining an accurate position estimate. Since LiDAR data is inherently high-dimensional and varies continuously with vehicle motion, robust and computationally efficient point cloud similarity metrics are essential. This section first formalizes the similarity problem and motivates the need for simplified metrics; it then presents the three methods evaluated in this study.

### 4.1. Problem Formulation

Let $P_{1}$ and $P_{2}$ be two point clouds. If it is possible to find a translation vector $v_{T}\in R^{3}$ and a rotation matrix R∈SO(3) such that $P_{1}$ and $RP_{2}+v_{T}$ significantly overlap, then $P_{1}$ and $P_{2}$ are considered similar, meaning they match. The main idea behind LiDAR-based localization is precisely this notion of point cloud similarity, and defining an unambiguous and computationally tractable measure for it is of critical importance.

Since rotations do not change the center of mass of a point cloud, without loss of generality we may assume that both point clouds have their centroids already aligned. Under this assumption the translation term $v_{T}$ can be dropped, and the similarity between $P_{1}$ and $P_{2}$ is defined as

(3) $$\mathrm{sim}\left(P_{1},P_{2}\right)=\min_{R\in SO(3)}\mathrm{dist}\left(P_{1},RP_{2}\right),$$

where dist(·,·) is a distance measure between two point clouds and SO(3) is the group of rotation matrices. In full generality, the minimization in Equation (3) is over the three-dimensional rotation group, which has three degrees of freedom. Throughout this work, however, we adopt a planar, yaw-dominant motion model that is appropriate for a vehicle driving on a road surface. Under this model the dominant rotation between two nearby scans is about the vertical axis (yaw), while pitch and roll vary only slightly between adjacent frames and are treated as negligible. With pitch and roll held fixed, the minimization over SO(3) reduces to a search over the two angular coordinates that describe the orientation of the scan in the horizontal plane, that is, a search over [0,2π)×[0,2π). The formulation that follows, and the three similarity metrics derived from it, should therefore be read as exact under yaw-only planar motion and as an approximation when pitch and roll are small but nonzero, as is the case for the near-level campus route used in this study.

Among the various point cloud distances available in the literature, including Hausdorff distance, one-sided Hausdorff distance, and Sinkhorn distance, this work adopts the Chamfer distance, defined as

(4) $$\mathrm{dist}\left(P_{1},P_{2}\right)=\frac{1}{2N_{1}}\sum_{i=0}^{N_{1}-1}{∥u_{i}-\mathrm{NN}\left(u_{i},P_{2}\right)∥}_{2}+\frac{1}{2N_{2}}\sum_{j=0}^{N_{2}-1}{∥v_{j}-\mathrm{NN}\left(v_{j},P_{1}\right)∥}_{2},$$

where $P_{1}=\left\{u_{0},\ldots ,u_{N_{1}-1}\right\}$, $P_{2}=\left\{v_{0},\ldots ,v_{N_{2}-1}\right\}$, and NN(x,P) denotes the nearest neighbor of *x* in the set *P*,

(5) $$\mathrm{NN}\left(x,P\right)=\arg\min_{y\in P}{∥x-y∥}_{2}.$$

While Equations (3)–(5) provide an ideal formulation of the problem, their direct application is computationally prohibitive in real-time settings. With point clouds containing approximately N=20,000 points, the nearest-neighbor computation requires a loop of size *N*, the Chamfer summation adds another loop of equal size, and the minimization over SO(3), under the planar yaw-dominant model described above, entails a two-dimensional search over [0,2π)×[0,2π). An autonomous vehicle traveling at speed has only a short time window available for localization. This motivates the three alternative similarity metrics described in the following subsections, each of which trades some geometric fidelity for substantially lower computational cost [10].

### 4.2. Fast Point Feature Histograms with KDTree

**Figure 6:**
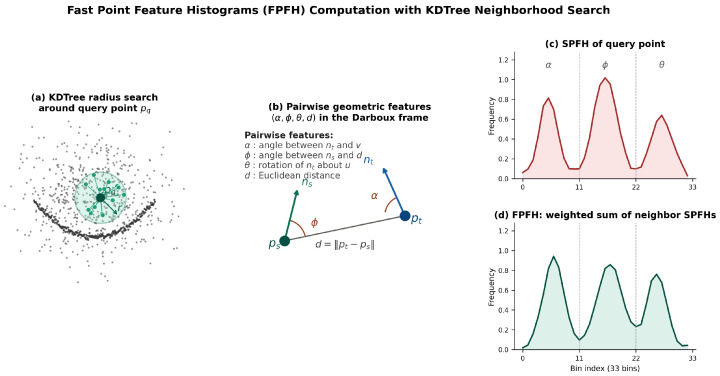
Visualization of FPFH descriptor computation. For each query point a spherical neighborhood is defined; the geometric relationships between the central point and its neighbors are encoded into a compact histogram descriptor.

Fast Point Feature Histograms (FPFH) is a widely used local geometric descriptor for point cloud processing, particularly in registration and localization tasks [5]. FPFH captures first-order geometric relationships between a point and its local neighborhood through three key features: the angular deviation between the surface normals of the central point and its neighbors, the difference in local curvature, and the Euclidean distance between the central point and its neighboring points. A visualization of the descriptor computation is shown in Figure 6.

In this study, FPFH features are computed using the Open3D library [19] function compute_fpfh_feature, where neighborhood points are retrieved within a specified radius using a KDTree. A KDTree [20] is a space-partitioning data structure that organizes points in a *k*-dimensional space by recursively splitting the dataset along alternating dimensions, enabling efficient nearest-neighbor queries that significantly reduce the number of distance computations required.

#### Similarity Metric

Let ${\mathrm{FPFH}}_{\mathrm{local}}=\left\{f_{l1},f_{l2},\ldots ,f_{lm}\right\}$ be the *m* FPFH descriptors extracted from the current LiDAR scan, and let ${\mathrm{FPFH}}_{\mathrm{global}}=\left\{g_{g1},g_{g2},\ldots ,g_{gn}\right\}$ be the *n* descriptors from the corresponding HD map entry. A KDTree is constructed over the local features, and for each local feature $f_{li}$ the *k* nearest neighbors in the global feature set are found,

(6) $$\mathrm{NN}\left(f_{li}\right)=\left\{g_{j1},g_{j2},\ldots ,g_{jk}\right\},\;i=1,2,\ldots ,m.$$

The Euclidean distance between each local feature and its *j*-th nearest global neighbor is

(7) $$d_{li,j}={∥f_{li}-g_{j}∥}_{2}.$$

Three statistical summaries are derived from these distances: the mean, maximum, and variance,

(8) $${\bar{d}}_{li}=\frac{1}{k}\sum_{j=1}^{k}d_{li,j},\;d_{li}^{\max}=\max_{j}\;d_{li,j},\;\sigma_{li}^{2}=\frac{1}{k}\sum_{j=1}^{k}{\left(d_{li,j}-{\bar{d}}_{li}\right)}^{2}.$$

The adjusted disparity score is the weighted combination

(9) $$\mathrm{adj}\_{\mathrm{score}}_{i}={\bar{d}}_{li}+0.5\times d_{li}^{\max}+0.3\times \sigma_{li}^{2}.$$

**Figure 7:**
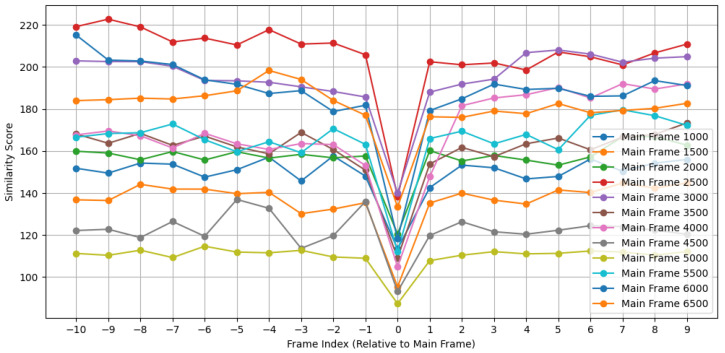
FPFH disparity scores as a function of frame index relative to the main frame, evaluated at twelve reference locations distributed along the driven route. All curves reach a minimum at Frame 0 and increase with distance. The relatively large spread between curves across different main frames reflects the sensitivity of FPFH descriptors to local geometric variation.

A lower value of $\mathrm{adj}\_{\mathrm{score}}_{i}$ indicates a higher similarity between the local scan and the corresponding HD map entry. The weighting coefficients 0.5 and 0.3 applied to the maximum and variance terms were set empirically and held fixed across all experiments, and the same values are reused in the Procrustes score of Section 4.3. Their role is to supplement the mean nearest-neighbor distance, which is the primary matching signal carrying unit weight, with two down-weighted secondary terms: the maximum distance, which penalizes the worst-matching descriptor and thus increases sensitivity to localized geometric discrepancies, and the variance, which penalizes inconsistent matching across the descriptor set. The maximum and variance terms are weighted below the mean so that they refine the ranking of candidate frames without overriding it. The relative ranking of candidates, and in particular the location of the minimum at Frame 0, is preserved under moderate changes to these two coefficients, so the localizability results reported in Section 6 do not depend on their precise values. Learning these weights from labeled localization outcomes, rather than fixing them, is identified as a direction for future work in Section 8.2. The behavior of this score across a window of ±10 frames around multiple reference frames is shown in Figure 7. All curves exhibit a clear V-shape with a minimum at Frame 0, confirming that the metric correctly identifies the reference frame as the most similar entry in the map. However, the inter-frame variation across different main frames is relatively large, reflecting the sensitivity of FPFH to local geometric differences.

### 4.3. Procrustes-Based Alignment

**Figure 8:**
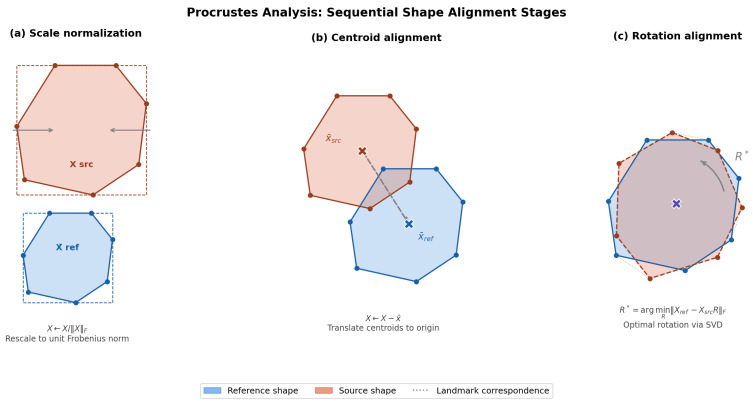
Illustration of the three Procrustes alignment stages: (**a**) scale normalization, (**b**) centroid alignment, and (**c**) rotation alignment. The method finds the optimal rigid transformation that minimizes the residual distance between two shapes.

Procrustes analysis is a statistical shape comparison method that measures similarity between two datasets by aligning them through a sequence of geometric transformations that minimize residual differences [21]. In the context of LiDAR point cloud comparison, the method provides a pose-invariant similarity evaluation that is well suited for localization, mapping, and trajectory estimation. The three sequential alignment stages, namely scale normalization (a), centroid alignment (b), and rotation alignment (c), are illustrated in Figure 8.

#### Similarity Metric

Let $P_{\mathrm{local}}=\left\{p_{1},\ldots ,p_{m}\right\}$ and $P_{\mathrm{global}}=\left\{q_{1},\ldots ,q_{n}\right\}$ denote the local LiDAR scan and the HD map reference, respectively. Both point clouds are normalized to an equal number of points (m=n). We note at the outset that classical Procrustes analysis presumes a known, ordered point-to-point correspondence between the two sets, so that the *i*-th point of one cloud is matched to the *i*-th point of the other. Two LiDAR scans acquired at nearby but distinct poses do not provide such an ordered correspondence, and normalizing the two clouds to a common cardinality does not by itself establish one. The procedure described below should therefore be understood as an approximate, correspondence-free variant of Procrustes alignment rather than as strict Procrustes analysis: it computes the optimal rigid rotation between the two centered point sets in closed form via singular value decomposition of their cross-covariance matrix, treating the index order produced by the normalization step as a working correspondence. This approximation is appropriate for the similarity-scoring task addressed here, where the goal is to produce a monotone disparity that is minimized when two scans originate from the same location, rather than to recover an exact registration between individually corresponding points. The empirical behavior of the resulting score, including its exact zero at Frame 0 and its smooth increase with frame distance, is examined in Section 6.1. The centroids are computed and both clouds are translated to the origin,

(10) $$C_{\mathrm{local}}=\frac{1}{m}\sum_{i=1}^{m}p_{i},\;C_{\mathrm{global}}=\frac{1}{n}\sum_{j=1}^{n}q_{j},$$

(11) $$P_{\mathrm{local}}^{\prime }=P_{\mathrm{local}}-C_{\mathrm{local}},\;P_{\mathrm{global}}^{\prime }=P_{\mathrm{global}}-C_{\mathrm{global}}.$$

The optimal rotation matrix *R* is found via Singular Value Decomposition of the cross-covariance matrix *H*,

(12) $$H={P_{\mathrm{local}}^{\prime }}^{T}P_{\mathrm{global}}^{\prime },\;U,S,V^{T}=\mathrm{SVD}\left(H\right),\;R=VU^{T}.$$

An optional scale factor normalizes differences in point cloud density,

(13) $$s=\frac{\mathrm{trace}(S)}{\sum_{i=1}^{m}{∥P_{\mathrm{local},i}^{\prime }∥}^{2}},\;P_{\mathrm{local}}^{\prime \prime }=sRP_{\mathrm{local}}^{\prime }.$$

Three statistical metrics are derived from the per-point alignment errors between the transformed local scan and the global reference,

(14) $$\begin{array}{cc} \bar{e} & =\frac{1}{m}\sum_{i=1}^{m}{∥P_{\mathrm{local},i}^{\prime \prime }-P_{\mathrm{global},i}^{\prime }∥}^{2}, \end{array}$$

(15) $$\begin{array}{cc} e_{\max} & =\max_{i}\;∥P_{\mathrm{local},i}^{\prime \prime }-P_{\mathrm{global},i}^{\prime }∥, \end{array}$$

(16) $$\begin{array}{cc} \sigma_{e}^{2} & =\frac{1}{m}\sum_{i=1}^{m}{\left(∥P_{\mathrm{local},i}^{\prime \prime }-P_{\mathrm{global},i}^{\prime }∥-\bar{e}\right)}^{2}. \end{array}$$

The adjusted Procrustes disparity score is

(17) $$\mathrm{adj}\_\mathrm{score}=\bar{e}+0.5\times e_{\max}+0.3\times \sigma_{e}^{2}.$$

**Figure 9:**
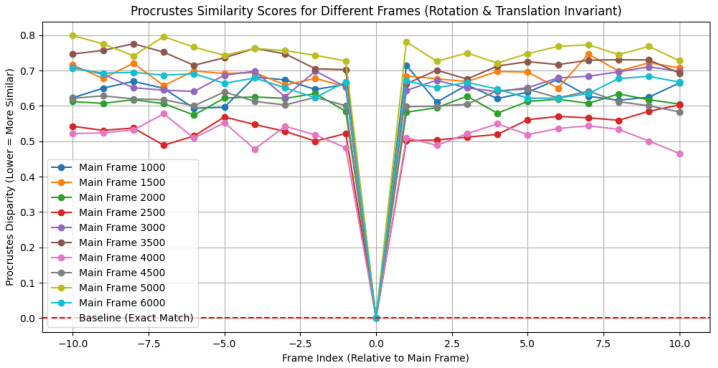
Procrustes disparity scores as a function of frame index relative to the main frame, evaluated at ten reference locations. All curves reach an exact minimum of zero at Frame 0 and increase consistently with distance. The tighter clustering and smoother profiles compared to FPFH reflect the rotation and translation invariance of the Procrustes alignment.

As with the FPFH metric, a lower score indicates better alignment, and the weighting coefficients 0.5 and 0.3 are the same empirically fixed values justified in Section 4.2. The behavior of this score across a ±10 frame window is shown in Figure 9. Compared to FPFH, the Procrustes disparity curves are notably smoother and more tightly clustered, reflecting the rotation and translation invariance of the alignment. All curves reach an exact minimum of zero at Frame 0, confirming the self-similarity property of the metric, and rise consistently as the frame distance increases.

### 4.4. Planar Projection Matching

**Figure 12:**
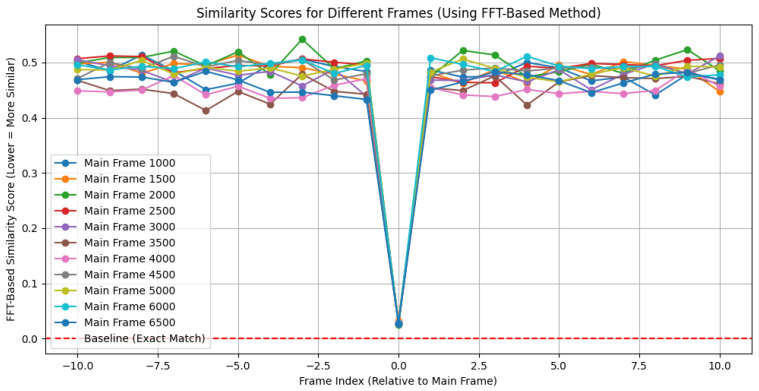
Planar projection disparity scores based on FFT cross-correlation, as a function of frame index relative to the main frame, evaluated at eleven reference locations. All curves reach a sharp minimum at Frame 0. The tight clustering across all main frames reflects the high consistency and stability of this metric compared to the FPFH and Procrustes methods.

The planar projection method addresses the computational burden of the full three-dimensional similarity formulation by reducing each point cloud to a compact two-dimensional representation before comparison [10]. The key observation is that, under the planar, yaw-dominant motion model adopted in Section 4.1, rotations of a three-dimensional point cloud correspond to circular translations in a suitable two-dimensional angular representation, allowing the minimization over SO(3) in Equation (3) to be replaced by an efficient cross-correlation operation. This correspondence is exact when the rotation between two scans is a pure yaw rotation about the vertical axis, since a yaw rotation shifts the azimuth coordinate of every point by a constant amount and leaves the elevation coordinate unchanged, which appears as a circular shift along one axis of the angular histogram. When pitch and roll are nonzero, the mapping is no longer an exact circular shift, because out-of-plane rotations couple the azimuth and elevation coordinates nonlinearly. For the small pitch and roll variations encountered on the near-level campus route, this coupling is minor and the circular-shift model remains a good approximation, as the empirical score profiles in Figure 12 confirm; for trajectories with substantial grade or banking, the approximation error would grow, as noted in the limitations of Section 7.4.2.

Each point cloud *P* is first expressed in spherical coordinates,

(18) $$P=\{\left(r_{k},\theta_{k},\phi_{k}\right):k=0,\ldots ,N-1\},$$

and the radius component is then discarded to obtain the reduced set of angular coordinates,

(19) $$Q=\{\left(\theta_{k},\phi_{k}\right):k=0,\ldots ,N-1\}.$$

**Figure 10:**
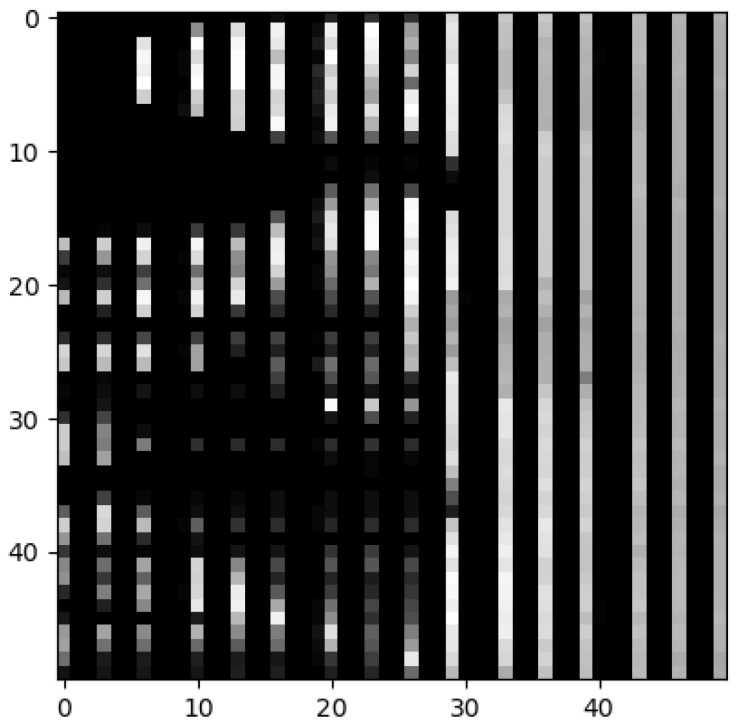
Image visualization of the two-dimensional angular histogram of a LiDAR point cloud after discarding the radius component. Each pixel intensity corresponds to the density of LiDAR returns at a given azimuth and elevation angle. Source: thesis.

**Figure 11:**
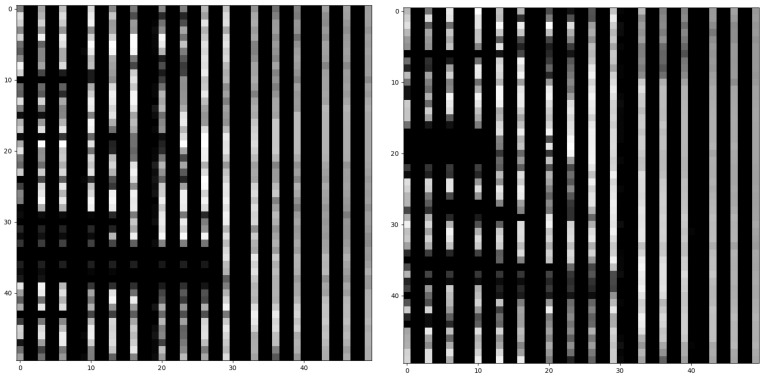
Two-dimensional histogram representations of two LiDAR point clouds captured five seconds apart (t=40 s, (**left**); t=45 s, (**right**)). The structural similarity between consecutive scans is clearly visible, confirming the suitability of the histogram representation for cross-correlation-based similarity scoring. Source: conference paper.

Dropping the radius introduces some loss of information but greatly reduces subsequent computational overhead. The set *Q* is binned into a b×b two-dimensional histogram *H*, where *b* is the number of bins, typically chosen in the range b=50 to b=100. This matrix can be visualized as a grayscale image whose pixel intensity reflects the density of LiDAR returns at each angular position. An example of this representation for a sample scan is shown in Figure 10, and side-by-side representations of two scans captured five seconds apart are shown in Figure 11 to illustrate the gradual structural evolution of the scene as the vehicle moves.

#### Similarity Metric

Given two point clouds $P_{1}$ and $P_{2}$ with angular histograms $H_{1}$ and $H_{2}$, we first define the maximum normalized circular cross-correlation over all possible row and column shifts,

(20) $$\rho \left(P_{1},P_{2}\right)=\frac{\max_{k,\;\ell }\sum_{i,j}H_{1}\left[i,j\right]\;H_{2}\left[i+k,\;j+\ell \right]}{\sqrt{\left(\sum_{i,j}H_{1}{\left[i,j\right]}^{2}\right)\left(\sum_{i,j}H_{2}{\left[i,j\right]}^{2}\right)}},$$

where all matrix indices are taken modulo *b*. The numerator is the two-dimensional circular cross-correlation maximized over all shifts, which accounts for yaw rotation under the planar motion model of Section 4.1; the denominator normalizes by the energy of each histogram so that ρ∈[0,1], with ρ=1 attained when a scan is correlated with itself. So that the planar projection score follows the same “lower is better” convention as the FPFH and Procrustes metrics, we define the planar projection disparity as

(21) $$\mathrm{disp}\left(P_{1},P_{2}\right)=1-\rho \left(P_{1},P_{2}\right).$$

This disparity equals zero for an exact self-match and increases toward one as two scans diverge, mirroring the exact-zero baseline of the Procrustes metric. This formulation accounts for yaw rotations of the point cloud, since a yaw rotation about the vertical axis corresponds to a circular shift in the angular histogram. The double summation in Equation (20) as a function of shifts *k* and *ℓ* is equivalent to a two-dimensional cross-correlation, which is computed efficiently via the Fast Fourier Transform, reducing computational complexity substantially compared to the direct Chamfer distance approach [10].

The behavior of the planar projection disparity score across a ±10 frame window is shown in Figure 12. As with the other two methods, all curves exhibit a clear V-shaped minimum at Frame 0. The planar projection scores are the most tightly clustered of the three methods, indicating high consistency across different reference locations and making this metric particularly well suited for stable, repeatable localization.

### 4.5. Summary

For consistency, all three methods are expressed throughout this paper as disparity scores under a single convention: a lower score indicates a better match, and a score of zero, attained exactly by the Procrustes and planar projection metrics, indicates a perfect self-match. The intermediate Euclidean distances between feature vectors in the FPFH method, and the normalized cross-correlation ρ in the planar projection method, are named according to their standard meanings, while “disparity score” refers specifically to the final lower-is-better quantity that each method feeds to the localization algorithm of Section 5. Table 1 summarizes the three similarity methods evaluated in this study, contrasting their underlying approach, key properties, and the form of the resulting disparity score.

## 5. Localization Framework

This section describes the recursive localization framework that ties together the HD map construction described in Section 3 and the three similarity metrics presented in Section 4. The framework provides a unified structure within which any of the three methods can be substituted as the similarity oracle, allowing their localization behavior to be compared under identical conditions.

### 5.1. Motivation and Overview

The localization problem addressed in this work can be stated as follows. Assume that the current GPS location of the vehicle is known at time t=k seconds. After one second the GPS signal is lost or unavailable, but a new LiDAR scan of the surrounding environment is acquired at the unknown location. The goal is to use this new scan, together with the HD map, to recover the vehicle’s updated position without relying on the GPS receiver.

The proposed approach is based on a two-dimensional binary search over the set of reachable GPS locations in the HD map. The key insight, established experimentally through the similarity score analyses presented in Section 4, is that all three similarity metrics produce a consistent and localizable signal: the similarity score between a query scan and a stored map scan is minimized when the two scans originate from the same location, and increases monotonically as the spatial separation between the two locations grows. This property, illustrated by the V-shaped curves in Figure 7, Figure 9 and Figure 12, makes it possible to identify the most likely vehicle location by finding the map entry whose stored scan yields the lowest disparity score against the current scan.

### 5.2. Recursive Localization Algorithm

The proposed localization algorithm is recursive and is designed around a one-second update cycle that would match the LiDAR frame rate in an online deployment. We emphasize that this one-second cycle is the target update rate of the intended online architecture, not a rate achieved by the present offline study; as quantified in Section 5.4, the per-pair computation times of all three metrics currently exceed what this cycle would allow. It is formalized in the following seven steps [10]:

**S1.**  Initialize with k=0 and a known current location $g_{k}\in R^{3}$, provided by the RTK-GPS receiver at the start of the localization session.
**S2.**  After one second, acquire a new LiDAR point cloud $P_{k+1}$ at the current (unknown) vehicle location.
**S3.**  Let $S_{k}=\left\{g_{\ell }^{\prime }:\ell =0,\ldots ,M-1\right\}$ be the set of all GPS locations in the HD map that are reachable from $g_{k}$ within one second given the vehicle’s maximum speed. This set is determined by querying the directed graph structure of the HD map for all nodes within a radius corresponding to the maximum distance the vehicle can travel in one second.
**S4.**  Let $P_{k}=\left\{P_{\ell }^{\prime }:\ell =0,\ldots ,M-1\right\}$ be the set of LiDAR point clouds stored in the HD map at the GPS locations in $S_{k}$. These are the candidate clouds against which the new scan $P_{k+1}$ will be compared.
**S5.**  Compute the similarity score between $P_{k+1}$ and each candidate cloud $P_{\ell }^{\prime }\in P_{k}$ using one of the three similarity metrics described in Section 4. Find the candidate cloud $P^{*}$ that achieves the minimum adjusted similarity score,
  (22) $$P^{*}=\arg\min_{P_{\ell }^{\prime }\in P_{k}}\;\mathrm{adj}\_\mathrm{score}\left(P_{k+1},\;P_{\ell }^{\prime }\right).$$
**S6.**  Update the estimated vehicle location $g_{k+1}$ as the GPS coordinate in the HD map associated with the best-matching cloud $P^{*}$,
  (23) $$g_{k+1}=g_{\ell^{*}}^{\prime },\;\mathrm{where}\;\ell^{*}=\arg\min_{\ell }\;\mathrm{adj}\_\mathrm{score}\left(P_{k+1},\;P_{\ell }^{\prime }\right).$$
**S7.**  Increment *k* by one and return to Step S2.

**Figure 13:**
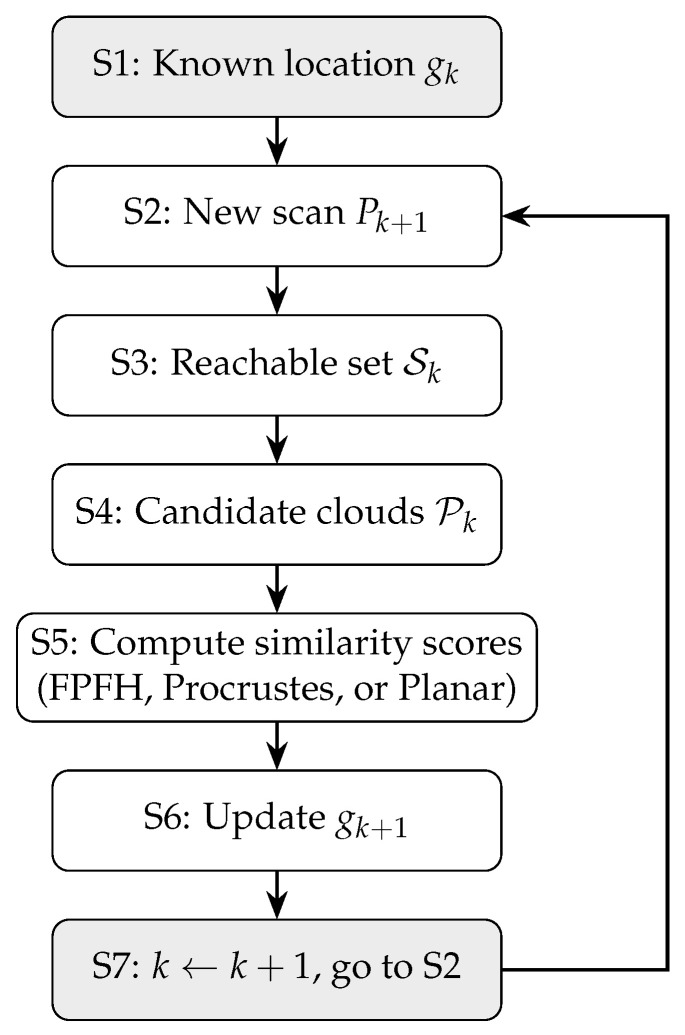
Schematic of the recursive localization algorithm (S1–S7). One cycle runs every second. Step S5 is the only step that varies across the three methods; all other steps are shared.

The algorithm terminates when the vehicle’s journey ends or when the GPS signal is restored. A schematic overview of one recursive update cycle is shown in Figure 13.

### 5.3. Integration of the Three Similarity Methods

The S1–S7 algorithm described above is designed as a method-agnostic framework. The only step that differs across the three similarity metrics is Step S5, where the adjusted similarity score is computed. Steps S1 through S4 and Steps S6 and S7 are identical regardless of which metric is used. This design allows the three methods to be evaluated under strictly identical conditions, isolating the effect of the similarity metric on the overall localization behavior.

When using FPFH with KDTree as the similarity oracle in Step S5, the adjusted score $\mathrm{adj}\_\mathrm{score}(P_{k+1},P_{\ell }^{\prime })$ is computed according to Equation (9). The FPFH descriptors of both the query scan and each candidate cloud must be computed before the score can be evaluated, which is the primary source of the high computational cost of this method.

When Procrustes-based alignment is used in Step S5, the adjusted score is computed according to Equation (17). Both the query scan and each candidate cloud are first normalized to have the same number of points, after which the centroids are aligned and the optimal rotation is found via SVD. Because the Procrustes alignment operates directly on the raw point coordinates without requiring descriptor extraction, it is substantially faster than FPFH.

When the planar projection method is used in Step S5, the adjusted score is computed as the planar projection disparity of Equation (21), that is, one minus the maximum normalized FFT cross-correlation of Equation (20). Each point cloud is first converted to its b×b angular histogram representation, and the disparity is then evaluated using the FFT. The absence of both descriptor extraction and iterative alignment makes this the fastest of the three methods while still producing a clear and reliable localization signal.

Table 2 summarizes how each method integrates into Step S5 of the algorithm, highlighting the computational operation performed and the representation required for each input point cloud.

### 5.4. Computational Considerations

The recursive nature of the algorithm means that every localization update requires evaluating $|S_{k}|=M$ similarity scores in Step S5, where *M* is the number of reachable map nodes within one second of travel. For a vehicle moving at moderate speed, *M* is typically in the range of tens to low hundreds of frames. The total computational cost of each update cycle therefore scales linearly with *M* and with the per-pair cost of the chosen similarity metric.

As shown in the experimental results in Section 6, the per-pair computation times differ dramatically across the three methods: FPFH requires an average of 1018.1 s per pair, making the full update cycle completely impractical for real-time use; Procrustes analysis requires an average of just 2.63 s per pair, a reduction of approximately 387 times; and the planar projection method requires an average of 11.6 s per pair, placing it between the other two in terms of speed while remaining the method of choice when a balance between structural fidelity and computational efficiency is required [10].

To make the consequence for online operation explicit, consider the total cost of a single update cycle, which is the per-pair cost multiplied by the number of candidate clouds *M*. Even for the fastest metric, Procrustes at 2.63 s per pair, a modest candidate set of M=30 already implies roughly 79 s per update cycle, and a larger set of M=100 implies more than four minutes; the planar projection method is slower still, and FPFH is slower by a further two orders of magnitude. In every case the per-cycle cost exceeds the one-second target by a wide margin. The recursive framework of this section should therefore be understood as the intended deployment architecture and as the structure under which the three metrics are compared on identical terms, rather than as a system that operates in real time at its present per-pair cost. The algorithmic and hardware directions that would be required to bring the per-cycle cost within the one-second budget, including candidate-set pruning, point cloud downsampling, and hardware acceleration, are discussed in Section 8.2.

## 6. Experimental Results

This section presents the experimental evaluation of the three similarity metrics within the localization framework described in Section 5. The evaluation covers two complementary aspects: the behavior of each similarity score as a function of spatial distance from a reference frame, and the computational cost of each method measured across five independent trials.

### 6.1. Similarity Score Evaluation

#### 6.1.1. Experimental Protocol

To assess the localizability of each similarity metric, a set of specific LiDAR point cloud frames were selected from the dataset as reference frames, referred to as main frames. For each main frame, the disparity score was computed between that frame and every neighboring frame within a window of ±10 frames, yielding a total of 21 comparison points per main frame, including the main frame itself and ten preceding and ten succeeding frames. This window corresponds to a spatial range of approximately ±10 m along the driven route at the vehicle’s operating speed. To ensure that the results are representative of the full dataset and not specific to any single location, the experiment was repeated for multiple main frames distributed across the entire 19,500-frame dataset, covering frame indices from 1000 to 6500.

A similarity metric is considered well suited for localization if it produces a consistent V-shaped score profile across all main frames, with a clear and unambiguous minimum at Frame 0. The sharper and more consistent the minimum, the more reliable the metric is expected to be in the recursive localization algorithm of Section 5.

#### 6.1.2. FPFH Score Profile

The FPFH disparity scores across the ±10 frame window for twelve main frames distributed along the route are shown in Figure 7. All curves exhibit a V-shaped profile, with the minimum score occurring at Frame 0 in each case, confirming that the FPFH metric correctly identifies the reference frame as the most geometrically similar entry in the HD map.

However, two notable characteristics distinguish the FPFH behavior from the other two methods. First, the absolute score values vary substantially across different main frames, spanning a range from approximately 90 to 220 depending on the location. This large inter-frame spread reflects the sensitivity of FPFH descriptors to local geometric variation along the route: areas with rich surface structure yield higher descriptor distances overall, while geometrically uniform areas produce lower scores. Second, the score profiles are noisier than those produced by the other two methods, with irregular fluctuations visible on both sides of the minimum. These fluctuations arise because FPFH operates on local neighborhoods and is therefore more susceptible to point cloud density variations and minor sensor noise. While the minimum at Frame 0 remains identifiable in all cases, the noisy profile makes the metric more challenging to use reliably in the localization algorithm when the similarity differences between neighboring frames are small.

#### 6.1.3. Procrustes Score Profile

The Procrustes disparity scores for ten main frames are shown in Figure 9. The results differ markedly from those of FPFH in two respects. First, all curves converge to an exact value of zero at Frame 0, reflecting the self-similarity property of the Procrustes alignment: when a point cloud is compared against itself, the optimal rigid transformation is the identity, and the residual alignment error is exactly zero. This clean zero baseline makes the minimum unambiguous and eliminates any ambiguity in score interpretation. Second, the curves are considerably smoother than those of FPFH, with far less inter-frame fluctuation on either side of the minimum. The spread between different main frames is also tighter, indicating that the Procrustes disparity is more stable across different environments along the route.

These properties are a direct consequence of the rotation and translation invariance of the Procrustes alignment. Because the method finds the globally optimal rigid transformation before computing the residual error, it is insensitive to the orientation of the vehicle at the time of scan acquisition, making it more robust to viewpoint differences between the query scan and the stored map entry. The main disadvantage of this stability, as discussed in Section 5.4, is its computational cost.

#### 6.1.4. Planar Projection Score Profile

The planar projection disparity scores for eleven main frames are shown in Figure 12. This method produces the tightest clustering of the three, with all main frame curves virtually overlapping across the entire ±10 frame window. Like the Procrustes method, the minimum occurs at Frame 0 in every case, but unlike FPFH, the minimum is sharp and consistent with very little noise on either side.

The tight clustering across different main frames indicates that the planar projection score is largely invariant to the specific location along the route, a desirable property for a localization metric since it means the score threshold for declaring a match does not need to be tuned differently for different parts of the map. The score rises steeply on both sides of the minimum, providing a strong gradient that makes the correct location easy to identify even in the presence of small perturbations.

The slightly less smooth profile compared to Procrustes is expected, since the planar projection discards the radius component of each point and operates on a binned histogram rather than on the raw coordinates. However, the loss of geometric fidelity is modest in practice, and the resulting score profiles remain clearly V-shaped and highly consistent across all tested locations.

#### 6.1.5. Cross-Method Comparison

Table 3 summarizes the key characteristics of the disparity score profiles produced by each method, based on the experimental observations described above.

### 6.2. Computational Time Comparison

#### 6.2.1. Experimental Protocol

To evaluate the computational efficiency of each similarity metric, the execution time required to compute a single pairwise disparity score was measured for each method over five independent trials. All experiments were run on the same hardware platform under identical conditions. The results are reported in Table 4.

#### 6.2.2. Analysis

The timing results reveal dramatic differences in computational efficiency across the three methods. FPFH with KDTree is by far the most expensive, with an average execution time of 1018.1 s per pairwise comparison, which is approximately 17 min. This cost is primarily driven by the FPFH descriptor extraction step, which requires computing a 33-dimensional histogram for every point in both clouds based on the geometric relationships within a local neighborhood, followed by a KDTree nearest-neighbor search over the full descriptor space. At this cost, even a single localization update in the S1–S7 algorithm would require evaluating scores against multiple candidate clouds, making real-time deployment entirely impractical with current hardware.

Procrustes analysis reduces this cost by a factor of approximately 387, with an average execution time of 2.63 s. This dramatic speedup is achieved by replacing descriptor extraction and nearest-neighbor search with a sequence of closed-form matrix operations: centroid subtraction, covariance matrix formation, and SVD. The five-trial variance is also notably high for Procrustes (ranging from 1.13 to 4.13 s), suggesting that the SVD computation time is sensitive to the numerical conditioning of the input point clouds, which varies depending on the geometric structure of the environment at each location.

The planar projection method occupies an intermediate position, with an average execution time of 11.6 s. This is 4.4 times slower than Procrustes but approximately 88 times faster than FPFH. The execution time is more consistent across trials (ranging from 9.6 to 13.7 s) than Procrustes, reflecting the more uniform nature of the FFT-based computation, whose cost depends primarily on the histogram size *b* rather than on the geometric properties of the point clouds.

**Figure 14:**
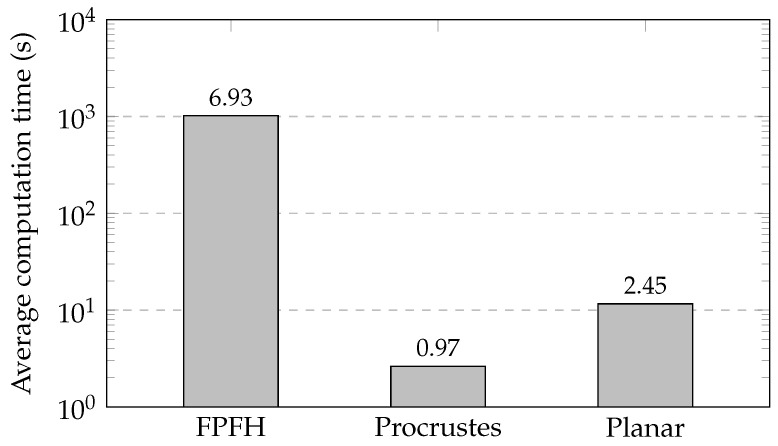
Average pairwise computation time for each method on a logarithmic scale. FPFH requires over 1000 s per comparison, while Procrustes and Planar Projection require 2.63 and 11.6 s respectively.

Figure 14 presents the average execution times on a logarithmic scale to make the orders-of-magnitude differences clearly visible. The speedup factors relative to FPFH are summarized in Table 5.

Taken together, the similarity score and computational time results establish a clear ordering across the three methods. Procrustes analysis offers the smoothest and most geometrically principled disparity profiles at the lowest per-pair computational cost of the three, making it the most promising of the three for an eventual online deployment, though as shown in Section 5.4 its per-cycle cost still exceeds the one-second target. The planar projection method provides similarly consistent V-shaped profiles at a moderate per-pair cost of 11.6 s, making it a reliable alternative among the offline metrics studied here. FPFH, while producing identifiable disparity minima, is the most expensive by a wide margin due to its extreme computational cost, and would require either hardware acceleration or significant algorithmic optimization before it could be deployed in an online AV system.

## 7. Discussion

The experimental results presented in Section 6 provide a consistent and interpretable picture of how the three similarity metrics behave under identical real-world conditions. This section synthesizes those findings into a broader discussion of the accuracy-versus-speed trade-off, the deployment contexts most suited to each method, and the limitations that must be considered before applying any of the three approaches in a production AV system.

### 7.1. Positioning Within GPS-Denied Localization

The assumption of a known initial GPS fix, made explicit in Section 7.4.5, places this work in a specific part of the broader GPS-denied localization landscape, and it is worth stating that position clearly. Two broad problem settings are usually distinguished in the literature. The first is global place recognition, sometimes called the kidnapped-vehicle or cold-start problem, in which the system must determine its location with no prior estimate, searching the entire map for a match. Learned global descriptors such as PointNetVLAD [14] and LPD-Net [15], and handcrafted global descriptors such as Scan Context [7], are designed primarily for this setting, where rotation invariance, viewpoint invariance, and the ability to retrieve a match from thousands of candidates are paramount. The second setting is local map-matching given a coarse prior, in which an approximate position is already available and the task is to refine or track it against a nearby portion of the map. The framework studied here belongs to this second setting: the recursive algorithm of Section 5 starts from a known fix and, at each step, searches only the small set of map nodes reachable within one update cycle. LiDAR-based localization under GNSS-denied conditions has likewise been demonstrated in constrained settings such as autonomous racing [22], where a known track map provides the prior. This is why our evaluation emphasizes the shape of the disparity profile in a narrow window around the true location, rather than global retrieval accuracy over the whole map. The known-initial-fix assumption is therefore not an incidental simplification but the defining characteristic that situates the work as a study of similarity metrics for the local map-matching step, and it explains why the metrics we compare are training-free geometric measures rather than the learned global descriptors that dominate the cold-start literature. Extending the framework toward cold-start operation would require pairing these local metrics with a global place-recognition front end, which we regard as complementary to, rather than in competition with, the present comparison.

### 7.2. Accuracy Versus Speed Trade-Off

**Figure 15:**
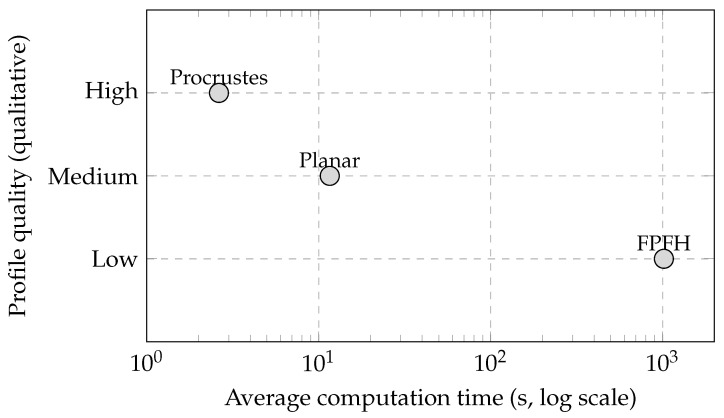
Qualitative accuracy-versus-speed trade-off for the three methods. Procrustes offers the best combination of profile quality and computation speed. Planar Projection is a strong intermediate option. FPFH provides identifiable but noisier profiles at a prohibitive computational cost.

The three methods occupy distinct and complementary positions along the accuracy-speed spectrum, as illustrated in Figure 15.

FPFH with KDTree sits at one extreme: it captures the richest local geometric information of the three methods, encoding angular deviations, curvature differences, and Euclidean distances into a 33-dimensional histogram per point. This richness is reflected in identifiable V-shaped similarity profiles, but at the cost of 1018.1 s per pairwise comparison, it is entirely impractical for real-time use. The high sensitivity of FPFH to local geometric structure also means that its absolute score values vary widely across different locations, complicating the design of a fixed similarity threshold for the localization algorithm.

Procrustes analysis occupies the opposite extreme in terms of speed. At 2.63 s per comparison on average, it is approximately 387 times faster than FPFH while producing smoother, more consistent score profiles. The exact zero at Frame 0 is a particularly valuable property: it means the self-similarity baseline is always precisely known, making it straightforward to define a reliable similarity threshold for the localization decision. The smoothness of the Procrustes disparity curves also reduces the risk of false minima in the localization step, which can occur when noisy score profiles produce spurious local dips at incorrect frame indices.

The planar projection method strikes a practical balance between the other two. Its execution time of 11.6 s is 4.4 times slower than Procrustes but 88 times faster than FPFH. Its score profiles are the most tightly clustered of the three methods, meaning that the similarity behavior is highly consistent regardless of where along the route the vehicle is located. This location-independence is advantageous in practice because it removes the need to recalibrate or retune the localization threshold as the vehicle moves through geometrically diverse parts of the map [10].

### 7.3. Method Selection by Deployment Context

The choice of similarity metric in a real-world AV localization system should be driven primarily by the available computational budget, the required update rate, and the geometric characteristics of the operating environment. Based on the experimental findings, the following deployment guidelines can be drawn.

#### 7.3.1. Geometrically Rich Urban Environments

Urban driving environments are characterized by dense, geometrically rich surroundings including buildings, parked vehicles, vegetation, and road infrastructure. These environments provide abundant local geometric features, which in principle favors descriptor-based methods such as FPFH. However, the computational cost of FPFH is the highest of the three methods by a wide margin, so among the metrics studied here it is the least suited to an eventual online deployment in this setting. Among the three, Procrustes is the most promising candidate for this context: it has the lowest per-pair cost and the smoothest, most well-defined score profiles, and its rotation and translation invariance is helpful in urban scenarios where the vehicle approaches intersections or navigates curved roads and the scan orientation changes rapidly between consecutive frames. We stress, however, that “most promising candidate” is a relative statement among the three offline metrics; as shown in Section 5.4, the per-cycle cost of Procrustes still exceeds the one-second target, so realizing online urban localization would require the candidate-set pruning, downsampling, or hardware acceleration discussed in Section 8.2 rather than the metric in its present form.

#### 7.3.2. Highway and Open-Road Driving

At highway speeds, the distance traveled between consecutive LiDAR frames is larger, meaning that the candidate set $S_{k}$ in Step S3 of the algorithm contains more reachable map nodes and the total localization cost per update scales accordingly. In this regime, among the three offline metrics the planar projection method is the more attractive candidate, because its consistent score profiles and predictable execution time, which depends primarily on the histogram size rather than on scene geometry, make it easier to bound the worst-case per-cycle cost once that cost has been brought within budget by the techniques of Section 8.2.

#### 7.3.3. Offline Map Matching and Post-Processing

In applications where computation is performed offline rather than in real time, such as batch map verification, dataset annotation, or forensic trajectory reconstruction, the computational cost of FPFH becomes less of a constraint. In these scenarios, FPFH may be preferred because its rich local descriptors capture fine-grained geometric differences that Procrustes and Planar Projection discard. The ability of FPFH to distinguish between geometrically similar but structurally distinct locations makes it particularly valuable for resolving ambiguities in maps where multiple locations have similar global shapes or angular distributions [5].

### 7.4. Limitations

#### 7.4.1. Sensitivity to Dynamic Environments

The most consequential limitation of the framework, affecting all three similarity metrics equally, is its reliance on a largely static environment. The HD map is built once from a single drive and is then treated as a fixed reference, so the localization signal depends on the assumption that the scene observed at query time matches the scene stored in the map. Real driving environments violate this assumption routinely rather than exceptionally: parked vehicles arrive and depart, construction changes the built environment, pedestrians and other road users move through the scene, and vegetation and seasonal conditions alter the returns from the same location over time. When such changes occur, the query scan and the stored map scan of the true location no longer match as closely, the disparity at Frame 0 is no longer the clean minimum observed in our experiments, and the localization algorithm may select an incorrect candidate. Because the three metrics all rest on scan-to-map similarity, none of them is immune to this effect; a metric that is more sensitive to fine geometric detail, such as FPFH, may in fact be more strongly disturbed by a local change than a metric that summarizes the scene more coarsely. We therefore regard sensitivity to environmental dynamics as a central constraint on the applicability of the present results, not as a minor caveat. The dataset used here was collected on a quiet campus route with relatively little dynamic traffic, which is favorable to the static-map assumption and should be kept in mind when interpreting the consistency of the score profiles reported in Section 6. Quantifying the degradation of each metric under controlled dynamic changes, and extending the framework with the incremental and multi-session map-update mechanisms discussed in Section 8.2.5, are necessary steps before the approach could be relied upon in environments where such dynamics are the norm.

It is useful to separate the kinds of dynamic change by how they affect the scan-to-map disparity. Transient moving objects, such as pedestrians or a single passing vehicle, perturb only a small fraction of the angular histogram or the local descriptor set, so their expected effect is a small upward shift of the disparity at the true location rather than a displacement of the minimum; the minimum at Frame 0 should remain identifiable as long as the static backdrop still dominates the scan. Sustained changes in traffic density, such as a lane of parked vehicles that is full in the map and empty at query time, alter a larger and spatially coherent portion of the scan, and we would expect these to be more damaging, since they bias the disparity in a consistent direction and can raise the disparity at the true location above that of a neighboring frame whose stored scan happens to resemble the changed scene more closely. The planar projection metric, which summarizes the whole scan into a single angular histogram, should be comparatively robust to small transient objects but sensitive to large coherent changes, whereas FPFH, which keys on fine local geometry, may be disturbed even by small changes near the sensor. Multi-session map inconsistency is a distinct concern that our single-session dataset cannot measure directly. When a map is assembled from drives recorded on different days, the same location may be represented by scans that differ in dynamic content, sensor pose, and even point density, and we would expect the disparity at the true location to inherit this variability, widening the score profiles and reducing the margin between the correct frame and its neighbors. Characterizing this degradation empirically, using repeated drives of the same route, is a necessary step that we identify as future work in Section 8.2.5; the present results, obtained from a single consistent session, should be read as an upper bound on the localizability achievable once multi-session variability is introduced.

#### 7.4.2. Planar Projection: Sensitivity to Flat and Open Environments

The planar projection method discards the radius component of each point before constructing the angular histogram, retaining only the azimuth and elevation angles of each LiDAR return. In environments that are geometrically flat or sparse, such as open parking lots, straight highway segments, or rural areas with few vertical structures, the angular histogram becomes dominated by the ground plane return and contains very little discriminative angular structure. In such cases, the 2D histogram representations of scans captured at different locations may be nearly identical, reducing the sharpness of the V-shaped minimum and potentially causing the localization algorithm to misidentify the correct frame. Increasing the histogram resolution by raising the bin count *b* can partially mitigate this issue but at the cost of increased memory and FFT computation time.

#### 7.4.3. FPFH: Sensitivity to Point Cloud Noise and Density Variation

FPFH descriptors are computed from local neighborhoods defined by a fixed-radius search, making them sensitive to variations in point cloud density. When the vehicle is closer to an object, the LiDAR returns from that object are denser and the resulting FPFH descriptors differ from those computed at a slightly greater distance, even if the underlying geometry is the same. This density sensitivity contributes to the large inter-frame spread observed in the FPFH score profiles and can cause incorrect matches in environments where the vehicle’s trajectory passes close to large structures such as buildings or parked vehicles. Additionally, FPFH relies on accurate surface normal estimation, which degrades in the presence of noise, sparse returns, or mixed-resolution regions near the boundary of the sensor’s field of view [5].

#### 7.4.4. Procrustes: Sensitivity to Point Count Normalization

The Procrustes alignment requires both point clouds to have the same number of points before the cross-covariance matrix can be formed. In this study, both clouds are normalized to a common size prior to alignment, introducing a dependence on the resampling strategy. Aggressive downsampling can remove geometrically important points and degrade alignment quality, while upsampling by interpolation introduces artificial points that do not correspond to real surfaces. The optimal point count is also environment-dependent and may not generalize across all deployment scenarios. Furthermore, as discussed in Section 4.3, the procedure is a correspondence-free approximation of Procrustes alignment; it effectively assumes that corresponding points exist in both clouds, which is not guaranteed when two scans are captured from significantly different positions or with different levels of occlusion.

#### 7.4.5. General Limitations of the Proposed Framework

Beyond the method-specific limitations described above, and beyond the sensitivity to dynamic environments discussed in Section 7.4.1, the localization framework itself has several further constraints that apply equally to all three similarity metrics. First, the framework assumes that the vehicle’s initial location is known from the GPS receiver, meaning it cannot be used for cold-start localization without an initial GPS fix. Second, the dataset used in this study was collected in a single session under favorable weather conditions, and the performance of all three methods under rain, fog, or dust, which directly affect LiDAR point density and noise levels, has not been evaluated [1]. Finally, all timing experiments were conducted on a single hardware platform; performance on embedded automotive-grade processors may differ significantly from the reported figures.

## 8. Conclusions and Future Work

### 8.1. Conclusions

This paper presented a unified high-definition map-based localization framework for autonomous vehicles and evaluated three point cloud similarity metrics within it under identical real-world experimental conditions. The framework was validated on a dataset of 19,500 time-synchronized LiDAR point clouds and RTK-GPS measurements, collected over a 30-min drive on the Florida Polytechnic University campus using a Velodyne VLP-16 sensor and a ublox ZED-F9P receiver. The HD map was constructed as a directed graph of GPS coordinates, each linked to a corresponding LiDAR scan, providing a complete and extensible reference database for the recursive localization algorithm.

The three similarity metrics evaluated, namely Fast Point Feature Histograms with KDTree-based matching, Procrustes-based alignment via singular value decomposition, and a planar projection method based on two-dimensional FFT cross-correlation, were each analyzed in terms of their similarity score profiles across a ±10 frame window at multiple reference locations, and their per-pair computation times across five independent trials.

The principal findings of the study can be summarized as follows. First, all three methods produce V-shaped similarity score profiles with a clear minimum at the reference frame, confirming that the similarity-based localization approach is feasible regardless of which metric is used. Second, the methods differ markedly in their score profile quality and computational cost. FPFH produces identifiable but noisy profiles with large inter-location variability, and its average execution time of 1018.1 s per pairwise comparison makes it impractical for real-time deployment. Procrustes analysis produces the smoothest and most consistent profiles, with an exact zero at the self-similarity baseline and tight clustering across different locations, at an average cost of just 2.63 s, representing a speedup of approximately 387 times over FPFH. The planar projection method produces the most location-invariant profiles of the three, with the tightest inter-frame clustering, at an average cost of 11.6 s, which is 4.4 times slower than Procrustes but 88 times faster than FPFH. Third, the three methods are complementary rather than interchangeable: among the offline metrics studied here, Procrustes is the most promising candidate for geometrically rich urban environments, planar projection is the more attractive candidate for highway and open-road scenarios, and FPFH is most valuable in offline applications such as batch map verification, where geometric precision takes priority over computational speed. In all three cases, the suitability is assessed relative to the other two metrics under the offline conditions of this study; bringing any of them to online operation would require the cost-reduction directions outlined in Section 8.2.

Taken together, these results demonstrate that the choice of similarity metric has a decisive impact on the practical viability of HD map-based localization, and that a single metric is unlikely to be optimal across all deployment contexts. The unified framework proposed in this work provides a principled basis for selecting and benchmarking similarity metrics, and the dataset and experimental protocol described here offer a reproducible foundation for future comparisons.

Beyond the per-metric findings, the comparison points to a broader insight for localization-system design. Across the three metrics, profile quality and location-invariance did not improve with geometric richness; if anything, the opposite held. The two metrics that summarize a scan more coarsely, Procrustes global alignment and the planar angular histogram, produced smoother and more location-invariant disparity profiles than FPFH, the richest and most locally detailed descriptor, which was also the noisiest and the most variable across locations as well as by far the most expensive. For the map-matching step studied here, where the goal is a clean, predictable minimum at the true location rather than a fine-grained description of local geometry, a globally-summarizing metric is therefore the more natural default. This suggests a design principle for the recursive framework: use an inexpensive, globally-summarizing metric as the primary matcher to obtain stable score profiles and a predictable per-cycle cost, and reserve a rich local descriptor such as FPFH for disambiguation only at the comparatively rare locations where the primary metric cannot separate adjacent candidates. This is precisely the adaptive-selection strategy outlined in Section 8.2.4, and the comparative results reported here give it an empirical basis: they indicate not only which metric to prefer by default, but also why the expensive metric is best held in reserve rather than used throughout.

### 8.2. Future Work

Several directions present themselves as natural extensions of this work, ranging from targeted algorithmic improvements to broader system-level enhancements.

#### 8.2.1. Hardware Acceleration for FPFH

The 1018.1-s average execution time of FPFH is not an inherent property of the descriptor itself but rather a consequence of its unoptimized CPU implementation. GPU-accelerated implementations of FPFH and KDTree nearest-neighbor search have been demonstrated in the robotics literature and could reduce this cost by one to two orders of magnitude [5]. Bringing the FPFH per-cycle cost below the one-second update threshold, through such acceleration together with the candidate-set pruning discussed below, would make it a viable option for online localization in geometrically rich urban environments, where its rich local descriptors are most likely to provide a genuine advantage over the other two methods.

#### 8.2.2. Reducing the Per-Cycle Localization Cost

The computational analysis in Section 5.4 showed that the cost of a single localization update is the per-pair similarity cost multiplied by the number of candidate clouds *M*, and that this per-cycle cost exceeds the one-second target for all three metrics at their present per-pair cost. Two complementary directions could narrow this gap independently of the choice of similarity metric. The first is candidate-set pruning: rather than scoring every reachable map node in $S_{k}$, a coarse-to-fine strategy could first reject implausible candidates using a cheap screening score, such as a low-resolution version of the planar histogram, and reserve the full similarity computation for a small shortlist, reducing the effective *M* by an order of magnitude or more. The second is point cloud downsampling: evaluating the similarity metrics on voxel-downsampled scans would lower the per-pair cost of all three methods, at the price of some geometric fidelity, and the trade-off between downsampling level and localizability is itself an open question worth characterizing. Combining aggressive pruning, downsampling, and the hardware acceleration discussed above is the most plausible route to bringing the recursive framework within a real-time budget, and quantifying the combined effect on both per-cycle cost and localization accuracy is a natural continuation of the present study.

#### 8.2.3. Learning-Based Similarity Metric Tuning

The weighting coefficients used in the adjusted similarity scores for all three methods, specifically the 0.5 multiplier on the maximum distance and the 0.3 multiplier on the variance, were set empirically in the present work and held fixed across all experiments. A natural extension is to learn these weights, or to replace the handcrafted score formulations entirely, using a neural network trained on labeled localization outcomes. Deep learning approaches such as PointNetVLAD [14] and LPD-Net [15] have demonstrated that end-to-end learned descriptors can outperform handcrafted ones on standard benchmarks. Integrating a lightweight learned similarity head into the localization framework, while keeping the HD map structure and recursive update algorithm unchanged, would allow the framework to adapt to new environments without requiring manual recalibration.

#### 8.2.4. Adaptive Metric Selection

The deployment context analysis presented in Section 7.3 suggests that no single metric is optimal across all environments and speed regimes. A practical improvement would be to implement an adaptive selection mechanism that switches between metrics at runtime based on environmental cues such as point cloud density, vehicle speed, and map coverage. For example, the system could default to Procrustes analysis in urban areas where dense geometry provides reliable global alignment, switch to planar projection on highway segments where consistent score profiles are more valuable than geometric precision, and invoke FPFH selectively at ambiguous locations where the Procrustes or planar projection scores are too similar between adjacent candidate frames to yield a confident localization decision.

#### 8.2.5. Multi-Session and Dynamic Map Updates

The HD map used in this study was constructed from a single data collection session and remains static throughout the localization experiments. In a real deployment, the environment changes over time due to construction, vegetation growth, parked vehicles, and seasonal variation. Extending the framework to support incremental map updates, where new LiDAR scans gradually update stored map entries rather than replacing them, would improve long-term localization reliability without requiring a full map rebuild. Multi-session map construction using scan merging algorithms [10] is a particularly promising direction, as it would allow the map to incorporate observations from multiple drives and thus build a richer and more robust representation of each location.

#### 8.2.6. Robustness to Adverse Weather Conditions

All experiments in this study were conducted under clear weather conditions. LiDAR sensors are known to be affected by rain, fog, and dust, which introduce noise and reduce the effective range and point density of each scan [1]. Evaluating the three similarity metrics under adverse weather conditions, and developing preprocessing steps such as rain removal, fog compensation, or adaptive normal estimation that improve robustness, would be a meaningful contribution toward production-ready deployment of the proposed framework.

#### 8.2.7. Multi-Sensor Fusion

The current framework uses LiDAR as the sole sensor for the localization step. Integrating additional modalities such as camera images, radar returns, or wheel odometry into the similarity score computation or into the HD map representation could improve localization robustness in situations where LiDAR alone is ambiguous, such as long featureless corridors or symmetric environments. The directed graph structure of the HD map is naturally extensible to multiple sensor modalities, as each node can be augmented with additional data without altering the structure of the recursive localization algorithm [2,4].

## Figures and Tables

**Table 1:** Summary of the three point cloud similarity methods evaluated in this study.

Method	Approach	Key Property	Score
FPFH with KDTree	Local feature descriptor	Rich local geometry	Lower is better
Procrustes Analysis	Global rigid alignment	Rotation, translation	Lower is better
		and scale invariant	
Planar Projection	2D angular histogram	Efficient FFT	Lower is better
	cross-correlation	cross-correlation	

**Table 2:** Integration of the three similarity methods into Step S5 of the localization algorithm.

Method	Representation	Core Operation in S5	Score Equation
FPFH with KDTree	FPFH descriptors per point	KDTree nearest-neighbor search	Equation (9)
Procrustes Analysis	Raw 3D coordinates (centered)	SVD of cross-covariance matrix	Equation (17)
Planar Projection	b×b angular histogram	FFT cross-correlation	Equation (21)

**Table 3:** Qualitative comparison of disparity score profile characteristics across the three methods, evaluated over the ±10 frame window at multiple main frames.

Method	Minimum at Frame 0	Profile Smoothness	Inter-Frame Spread	Zero Baseline
FPFH with KDTree	Yes, identifiable	Low (noisy)	High	No
Procrustes Analysis	Yes, exact zero	High (smooth)	Low	Yes
Planar Projection	Yes, sharp	Medium	Very low	No

**Table 4:** Pairwise disparity score computation time in seconds for each method across five independent trials, with averages. Source: thesis [10].

Trial	FPFH (s)	Procrustes (s)	Planar Projection (s)
1	1014.2	4.13	10.5
2	1020.2	1.13	11.6
3	1016.7	2.98	9.6
4	1020.8	3.24	13.7
5	1018.6	1.67	12.6
**Average**	**1018.1**	**2.63**	**11.6**

**Table 5:** Speedup factors relative to FPFH for the Procrustes and Planar Projection methods.

Method	Avg. Time (s)	Speedup vs. FPFH
FPFH with KDTree	1018.1	1× (baseline)
Planar Projection	11.6	≈88×
Procrustes Analysis	2.63	≈387×

## Data Availability

The original contributions presented in this study are included in the article. Further inquiries can be directed to the corresponding author.
